# Development of an algorithm to identify small cell lung cancer patients in claims databases

**DOI:** 10.3389/fonc.2024.1358562

**Published:** 2024-08-15

**Authors:** Mark D. Danese, Akhila Balasubramanian, D. Gwyn Bebb, Xerxes Pundole

**Affiliations:** ^1^ Outcomes Insights, Inc., United States, Calabasas, CA, United States; ^2^ Amgen Inc., Thousand Oaks, CA, United States

**Keywords:** small cell lung cancer, algorithm, claims, Medicare, antineoplastic therapy

## Abstract

**Introduction:**

The treatment landscape of small cell lung cancer (SCLC) is evolving. Evidence generated from administrative claims is needed to characterize real-world SCLC patients. However, the current ICD-10 coding system cannot distinguish SCLC from non-small cell lung cancer (NSCLC). We developed and estimated the accuracy of an algorithm to identify SCLC in claims-only databases.

**Methods:**

We performed a cross-sectional study of lung cancer patients diagnosed from 2016-2017 using the Surveillance, Epidemiology and End Results (SEER), linked with Medicare database. The analysis included two phases – data exploration (utilizing a 25% random sample) and data validation (remaining 75% sample). The SEER definition of SCLC and NSCLC were used as the gold standard. Claims-based algorithms were identified and evaluated for their sensitivity, specificity, positive predictive value (PPV), and negative predictive value (NPV).

**Results:**

The eligible cohort included 31,912 lung cancer patients. The mean age was 76.3 years, 44.6% were male, with 9.4% having SCLC and 90.6% identified as NSCLC using SEER. The exploration analysis identified potential algorithms based on treatment data. In the validation analysis of 7,438 lung cancer patients who received systemic treatment in the outpatient setting, an etoposide-based algorithm (etoposide use in 180 days following lung cancer diagnosis) to identify SCLC showed: sensitivity 95%, specificity 95%, PPV 82% and NPV 99%.

**Discussion:**

An etoposide treatment-based algorithm showed good accuracy in identifying SCLC patients. Such algorithms can facilitate analyses of treatment patterns, outcomes, healthcare resource and costs among treated SCLC patients, thereby bolstering the evidence-base for best patient care.

## 1 Introduction

Lung cancer is one of the most common cancers diagnosed in the United States (US) with an estimated 237,000 cases that were diagnosed in 2022 (1). Approximately 53% of lung cancer patients are male, and 69% of all patients are diagnosed at age 65 or older (2). There are two main types of lung cancer, non-small cell lung cancer (NSCLC) and small cell lung cancer (SCLC); the latter accounts for approximately 10% to 15% of all lung cancers (3). SCLC is an aggressive neuroendocrine carcinoma with a high risk of relapse and a low 5-year survival of <10% (4, 5). The treatment landscape in SCLC has recently been evolving with the approval of immunotherapies and many others under investigation.

With the evolving treatment paradigm in SCLC, evidence generated from real-world data sources such as electronic health records, administrative claims, and registries are needed to complement findings from trials, to generate evidence in the post-marketing setting and address gaps not evaluable in a trial setting (6, 7). Such data can be leveraged to characterize treatment patterns, measure outcomes, and estimate health resource utilization and cancer-related costs with the uptake of newly approved therapies.

In such secondary data analyses, specifically when leveraging administrative claims databases, the International Classification of Diseases (ICD), Ninth Revision, Clinical Modification (ICD-9-CM) or Tenth Revision, (ICD-10-CM) are typically used to identify patients with a specific tumor type. Unfortunately, the ICD system lacks specificity to distinguish the histological subtypes of lung cancer. Since the ICD coding system does not distinguish between SCLC and NSCLC, this distinction must be made using other data elements available in claims data sets. Furthermore, because there is little in the way of coding to distinguish between SCLC and NSCLC other than procedures and systemic therapies, the distinction can only be reliably made based on resources used in diagnosing and treating patients.

There are at least two key US-based studies that used claims-based algorithms to identify SCLC or NSCLC. In 2008, Sheng et al. developed an algorithm to distinguish SCLC cases from all lung cancer in administrative claims databases (8). They used American Cancer Society (ACS) and National Comprehensive Cancer Network (NCCN) treatment guidelines and clinical expertise to identify SCLC based on therapy. Although modified by other authors to reflect changes in treatment patterns, the algorithm was not validated.

In 2017, Turner, et al. updated a modified version of the Sheng SCLC algorithm to include first-line treatments and test recommendations for patients with NSCLC and SCLC according to 2015 ACS and 2016 NCCN guidelines (9). The validation was performed using the HealthCore Integrated Research Environment Oncology clinical data linked to the HealthCore Integrated Research Database (HIRD). Although the authors reported their results for NSCLC, the estimates for SCLC are readily calculated by switching sensitivity and specificity and switching positive and negative predictive values (NPV and PPV) to yield a sensitivity of 81.1%, specificity of 94.8%, PPV of 79.6%, and an NPV of 95.3%.

The Turner study used commercial claims data and had an average age of approximately 60 years, suggesting that it was most relevant for younger lung cancer patients who constitute a minority of all lung cancer patients [i.e., approximately 31% of patients are less than 65 years old at diagnosis (2)]. Therefore, our primary objective was to create potential algorithms for identifying SCLC using relevant interventions, available in claims-only databases, and to estimate the accuracy of algorithms by estimating the sensitivity, specificity, positive predictive value (PPV), and negative value (NPV) of these algorithms compared to the Surveillance, Epidemiology and End Results (SEER) histology information.

## 2 Materials and methods

### 2.1 Study design

This cross-sectional study used data from the National Cancer Institute (NCI) SEER cancer registry linked with Medicare enrollment and claims data from 1 January 2015 through 31 December 2017 (10). It was conducted among all patients diagnosed with lung cancer as defined in the SEER data.

### 2.2 Study data source

This study utilized a limited dataset from the NCI that was created by linking data from the SEER-18 registry with Medicare enrollment and claims. The SEER-18 registry covers approximately 28% of the US population (11). The SEER program collects data on incident cancer for persons diagnosed with a new primary cancer who reside in one of the SEER geographic areas. The SEER data include patient demographic information as well as tumor characteristics (e.g., stage, grade, and histology). Medicare is a US program that provides health insurance for 97% of individuals age 65 years and over. Medicare coverage is also provided for people with end-stage renal disease or a qualifying disability independent of age.

In this study, linked Medicare claims for calendar years 2016 to 2017 were used to identify diagnoses and procedures, including medications, surgery, and radiation. Claims records used in this study originated from the hospital facility, outpatient facility, hospice, physician, home health, durable medical equipment, and Part D (prescription drugs) files, provided by Medicare.

### 2.3 Study observation period

The study index date was the first day of the month of lung cancer diagnosis as defined by the SEER program. The study observation period began 12 months prior to the index date. All patient observation periods continued until the occurrence of the first of the following events: 180 days after diagnosis, end of continuous enrollment in fee-for-service Medicare (Part A, Part B, and Part D), or death.

Since this study was effectively cross-sectional, there was no clear distinction between baseline and follow-up for most analyses. However, the 1-year period prior to the first lung cancer diagnosis was used to describe the baseline demographic and clinical characteristics of the lung cancer cohort. Also, the 180-day period after the first lung cancer diagnosis was used to identify utilization relevant for distinguishing between SCLC and NSCLC.

### 2.4 Study inclusion and exclusion criteria

Patients who met all the following criteria were included. They must have been diagnosed with lung cancer between January 1, 2016, and December 31, 2017 in the Medicare claims (see **Supplementary Materials** for codes). They must have been age ≥ 66 years at the time of diagnosis to allow for 12 months of pre-diagnosis history. In a sensitivity analysis patients age ≤ 65 years were included. They must have had at least 12 months of Medicare Part A, Part B, and Part D coverage and no HMO coverage prior to diagnosis. There were no exclusion criteria applied for this study.

### 2.5 Study variables

The cohort was characterized using information as close to the lung cancer diagnosis date as possible for the following variables: age, sex, race, histology, and stage (using the Derived SEER Combined Stage and SEER Historic Stage variables).

Utilization of health care services, including outpatient systemic therapy, was identified using Healthcare Common Procedure Coding System (HCPCS) codes and Current Procedural Terminology (CPT) codes. In addition, National Drug Codes (NDC) for oral therapies with intravenous equivalents were identified using information from the Durable Medical Equipment and Part D files.

Utilization was captured in the 180-day period after lung cancer diagnosis. For a sensitivity analysis, the period 30 days before lung cancer diagnosis was also used to capture utilization.

SCLC was identified from the SEER data by using International Classification of Disease for Oncology (ICD-O) histology codes for small cell (8002, 8041, 8042, 8043, 8044, 8045) (12). All other patients were considered to have NSCLC. In the very rare event that there was more than 1 SEER record for malignant lung cancer, the SEER lung cancer histology record closest to the Medicare claim-based month and year of diagnosis was used. (Note that SEER only provides the month and year of diagnosis.)

The SEER definition of SCLC and NSCLC was the gold standard, and claims-based algorithms based on resource utilization were evaluated for their sensitivity, specificity, PPV, and NPV.

### 2.6 Analyses

Our analyses were conducted in two phases: exploration and validation. Exploration was carried out with a 25% random sample of all lung cancer patients, and validation was performed on the remaining 75% of the sample. Analyses included sensitivity, specificity, PPV and NPV (13).

Based on the fact that other published algorithms relied on resource utilization (primarily treatments) to identify SCLC, and based on our initial exploration of diagnosis codes and procedure codes in the lung cancer data, we found few codes to distinguish SCLC from NSCLC except for differences in health resource utilization. As a result, we conducted our analyses in two lung cancer cohorts: the full cohort of all diagnosed lung cancer patients and the subset that received at least one systemic therapy in the outpatient setting within 180 days from diagnosis (i.e., “systemic therapy subset”).

#### 2.6.1 Exploration sample

In the 25% exploration sample, we first identified all HCPCS/CPT codes that distinguished between SCLC and NSCLC. Because NSCLC represents over 80% to 85% of all lung cancers, factors that are more likely to occur in NSCLC are less useful for discriminating between SCLC and NSCLC unless the difference in utilization is very large. To identify potentially useful HCPCS/CPT codes, we identified all codes where the difference between the SCLC and NSCLC populations in the proportions using the code was at least 20%. This threshold was selected based on inspecting the distribution of differences among all HCPCS/CPT codes in the data, as well as by inspecting codes that did not meet this threshold to ensure that we were not missing any important codes. Because of the large disparity in prevalence between SCLC and NSCLC, we paid particular attention to codes that were common in SCLC and uncommon in NSCLC.

Next, we identified all codes that could potentially be used to reduce false positives for any algorithm. Since false positives represent NSCLC patients erroneously classified as SCLC, codes that are specific to NSCLC are most useful. We operationalized this as codes that were present in <5% of the SCLC population and >10% of the NSCLC population to ensure that we would not miss any important codes.

Similarly, we identified all codes that could potentially be used to reduce false negatives for any algorithm. Since false negatives represent SCLC patients erroneously classified as NSCLC, codes that are specific to SCLC are most useful. We operationalized this as codes that were present in >20% of the SCLC population and <5% of the NSCLC population.

Because systemic therapies can have many different codes, using individual codes could be misleading. Therefore, we also tested algorithms for systemic therapies in the exploration sample using all relevant codes for each therapy, instead of using individual codes.

Based on the results in the two 25% samples (diagnosed patients and treated patients), we selected relevant HCPCS/CPT codes for further evaluation in identifying patients with SCLC. We also included algorithms for all systemic therapy agents because etoposide was the most notable intervention that distinguished SCLC from NSCLC. Finally, because etoposide plus platinum is the most common first-line therapy in SCLC, we included a definition of all platinum agents for further evaluation.

#### 2.6.2 Validation sample

Using the 75% validation sample, we estimated the sensitivity, specificity, PPV and NPV for all algorithms selected in the exploration sample, both for all diagnosed patients and for the subset who received systemic therapy within 180 days of lung cancer diagnosis.

#### 2.6.3 Sensitivity analyses

We conducted two sensitivity analyses in the overall diagnosed patient population and the treated patient population. For the first, we included HCPCS/CPT codes from the 30-day period prior to diagnosis and re-ran the analysis code to evaluate the best algorithms from the validation analyses. Second, we evaluated the best algorithms from the validation analyses in the age 18-65 population.

#### 2.6.4 Data transformations and analyses

The raw SEER and Medicare data were decrypted and loaded onto a server. Using R, the raw data were converted to a standardized format [Generalized Data Model (14)] and then moved into a PostgreSQL database. Jigsaw software (Outcomes Insights, Inc., Calabasas, CA) was used to extract the analysis-ready data sets from the raw data. The analyses were conducted using R (version 4.1.3) (15).

Due to NCI privacy requirements, counts shown in tables that are <11, or that can be calculated and known to be <11, cannot be shown and are marked with “NR” for “Not Reportable”.

## 3 Results

**Figure 1:**
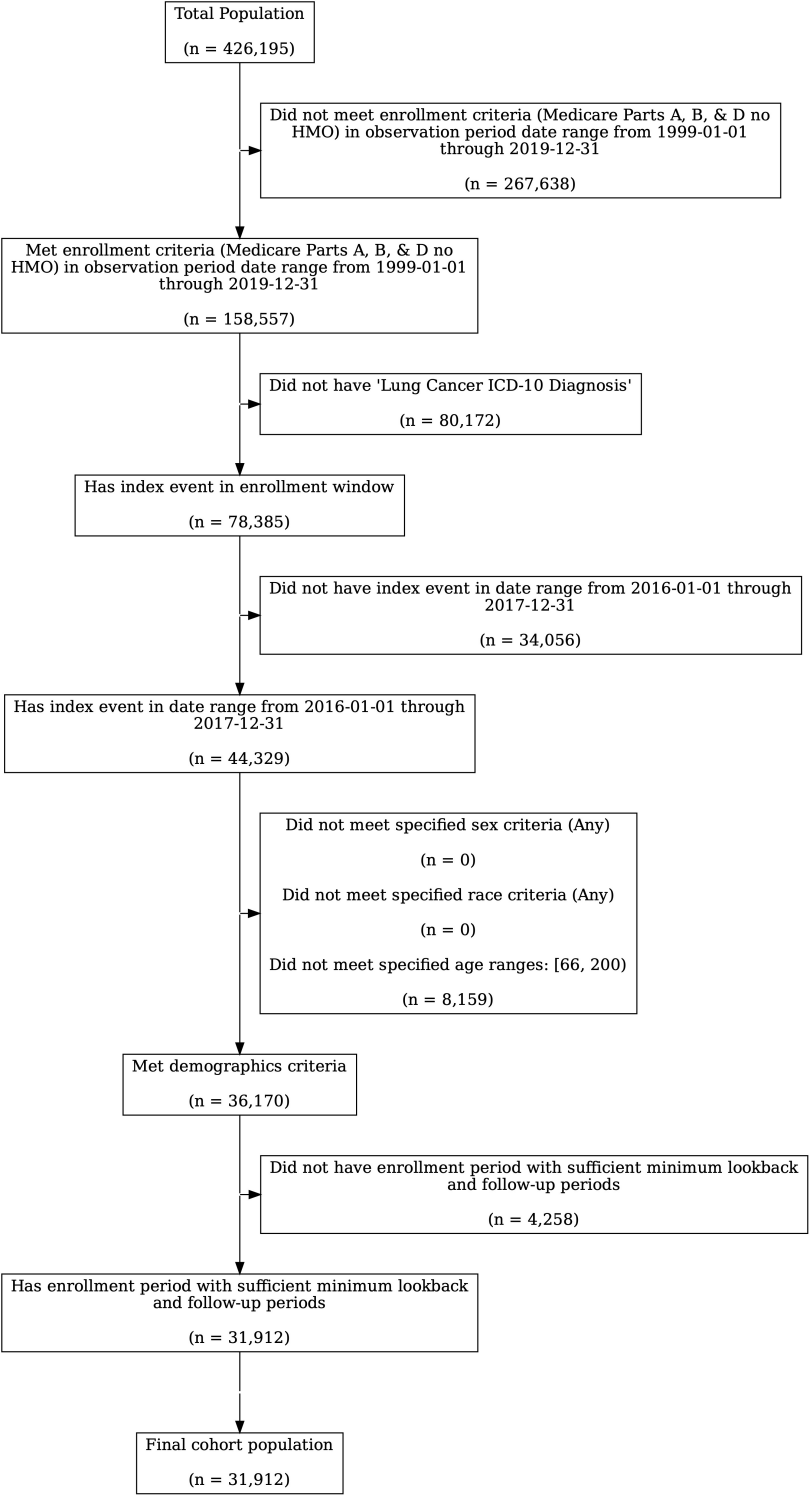
Cohort Creation Process. Note that the starting population for lung cancer patients included all patients diagnosed between 2010 and 2017 in SEER who were enrolled in Medicare.

The total SEER lung cancer population that met the Medicare Part A, B, and D enrollment requirements and had an ICD-10 diagnosis code for lung cancer in 2016 or 2017 was 44,329 (**Figure 1**). Of these, 36,170 (82%) met the age inclusion criterion, of whom 88% met the minimum 12-month lookback period requirement, resulting in a final cohort of 31,912 lung cancer patients. The process of creating the final cohort is described in **Figure 1**. This cohort included lung cancer patients treated with outpatient systemic therapy as well as those who were not. The 25% exploration sample had 10,006 lung cancer patients, of whom 2,568 were treated with systemic therapy within 180 days of diagnosis. The 75% validation sample had 23,934 lung cancer patients of whom 7,438 were treated with systemic therapy within 180 days.

The following summarizes the essential baseline demographic and clinical characteristics of the full cohort (**Table 1**). The mean age was 76.3 years, 44.6% were male, 9.4% were identified by SEER as having SCLC while 90.6% were identified as having NSCLC, and 41.7% were diagnosed with distant disease, 23.7% were diagnosed with regional disease and 34.1% were diagnosed with local disease. See **Supplementary Table 1** for the summary of baseline the demographic and clinical characteristics of the exploration and validation samples in both the full cohort and the systemic therapy subset.

**Table 1:** Population characteristics for lung cancer.

Variable	Full Cohort	Systemic Therapy Subset
	N = 31,912	N = 10,006
	% (N) or Mean (SD)	% (N) or Mean (SD)
Age at Diagnosis		
Age (years)	76.3 (7.0)	74.5 (6.0)
Age 66-69	18.9% (6,023)	24.1% (2,407)
Age 70-74	26.9% (8,594)	31.5% (3,150)
Age 75-79	23.9% (7,626)	24.6% (2,464)
Age 80-84	16.0% (5,095)	12.9% (1,291)
Age 85+	14.3% (4,574)	6.9% (694)
Sex and Race Categories		
Male	44.6% (14,248)	48.1% (4,814)
White	89.8% (28,646)	89.0% (8,910)
Black	5.8% (1,839)	5.8% (582)
Asian	3.7% (1,185)	4.4% (438)
Other	0.8% (242)	0.8% (76)
Marital Status		
Married	33.7% (10,747)	39.4% (3,943)
Widowed	15.1% (4,819)	12.6% (1,262)
Single/Divorced	14.7% (4,704)	14.8% (1,477)
Unknown	3.1% (1,004)	3.0% (299)
Missing	33.3% (10,638)	30.2% (3,025)
Histology		
Small Cell	9.4% (3,000)	17.6% (1,762)
Non-Small Cell	90.6% (28,912)	82.4% (8,244)
SEER Stage		
In Situ	0.5% (149)	NR
Local	34.1% (10,333)	9.6% (943)
Regional	23.7% (7,194)	32.3% (3,171)
Distant	41.7% (12,627)	58.0% (5,699)
Missing	5.0% (1,609)	NR
AJCC Stage		
In Situ	0.5% (111)	NR
Stage 1	33.0% (6,955)	7.5% (533)
Stage 2	7.7% (1,628)	9.3% (662)
Stage 3	18.1% (3,824)	29.5% (2,107)
Stage 4	40.7% (8,582)	53.7% (3,829)
Missing	33.9% (10,812)	NR
Year of Diagnosis		
2016	57.3% (18,276)	51.2% (5,124)
2017	42.7% (13,636)	48.8% (4,882)
Percent Poverty in Census Tract		
Poverty 0-4%	21.0% (6,691)	20.9% (2,092)
Poverty 5-9%	24.7% (7,882)	24.8% (2,485)
Poverty 10-19%	25.5% (8,143)	26.3% (2,629)
Poverty 20%+	17.4% (5,559)	16.9% (1,696)
Missing	11.4% (3,637)	11.0% (1,104)
Metropolitan Status of County		
Metro Area ≥ 1 Million Pop.	52.8% (16,843)	52.2% (5,226)
Metro Area < 1 Million Pop.	25.3% (8,082)	26.9% (2,688)
Adjacent to Metro Area	9.1% (2,898)	9.5% (947)
Not Adjacent to Metro Area	6.2% (1,968)	6.3% (634)
Missing	6.6% (2,121)	5.1% (511)

NR, not reportable due to NCI privacy rules that prevent reporting cell sizes < 11 or counts that can be used to calculate cell sizes < 11.

### 3.1 Exploration results

Using the 25% exploration sample, we evaluated all individual HCPCS or CPT codes in the lung cancer population to identify codes that were substantially more common in one subtype than the other. The largest difference in utilization between SCLC and NSCLC was for etoposide, which was used by 58% of diagnosed SCLC patients and 1.5% of NSCLC patients. The other factor that was potentially useful was pegfilgrastim use (40% of SCLC and 6% of NSCLC). Other interventions such as fosaprepitant (29% of SCLC and 7.7% of NSCLC) were less useful because they were too commonly used in NSCLC. In that example, there were 554 NSCLC patients who used fosaprepitant compared to only 213 SCLC patients who used it, despite the substantially more frequent use of fosaprepitant in SCLC compared to in NSCLC patients.

We identified one code that was potentially useful for reducing false positives: the use of EGFR testing (CPT code 81235; <1% of SCLC and 10% of NSCLC). We identified no codes that might reduce false negatives. Other interventions were not judged to be useful enough based on either their prevalence or their clinical relevance. Similar results were identified in the treated patient subset (data not shown).

We also explored the use of various systemic therapy agents. The utilization patterns of all systemic therapies in the exploration sample confirmed that etoposide was the most useful way to identify SCLC patients (sensitivity = 59%, specificity = 98%, PPV = 79% and NPV = 96%; **Table 2**). No other agents were better markers of SCLC. However, three other agents had high specificity, high PPV, and high NPV but low sensitivity and low utilization (**Table 2**): ipilumumab (PPV = 71% and NPV = 91%), irinotecan (PPV = 78% and NPV = 91%), and topotecan (PPV = 85% and NPV = 91%). Note that the high NPV in these 3 cases is caused by the low prevalence of SCLC.

**Table 2:** Exploratory results for selected algorithms for the full cohort and the systemic therapy subset.

Algorithm	True Positive	False Positive	True Negative	False Negative	Sensitivity	Specificity	PPV	NPV
Full Cohort (N = 7,978)								
**Carboplatin**	368	1,410	5,843	357	0.508	0.806	0.207	0.942
**Cisplatin**	92	228	7,025	633	0.127	0.969	0.288	0.917
**Etoposide**	431	116	7,137	294	0.594	0.984	0.788	0.960
**G-CSF**	319	612	1,509	128	0.714	0.711	0.343	0.922
**Ipilumumab**	NR	NR	NR	NR	< 0.02	> 0.99	0.714	0.910
**Irinotecan**	NR	NR	NR	NR	< 0.02	> 0.99	0.778	0.911
**Nivolumab**	17	157	7,096	708	0.023	0.978	0.098	0.909
**Pegfilgrastim**	297	471	6,782	428	0.410	0.935	0.387	0.941
**Topotecan**	NR	NR	NR	NR	< 0.02	> 0.99	0.846	0.910
**Platinum**	441	1,603	5,650	284	0.608	0.779	0.216	0.952
**Etoposide or Pegfilgrastim**	434	528	6,725	291	0.599	0.927	0.451	0.959
**Etoposide or G-CSF**	435	659	1,462	12	0.973	0.689	0.398	0.992
**Etoposide or Irinotecan or Topotecan**	438	120	7,133	287	0.604	0.983	0.785	0.961
**Etoposide and no reported EGFR testing**	428	104	7,149	297	0.590	0.986	0.805	0.960
Systemic Therapy Subset (N = 2,568)								
**Carboplatin**	368	1,410	711	79	0.823	0.335	0.207	0.900
**Cisplatin**	92	228	1,893	355	0.206	0.893	0.288	0.842
**Etoposide**	431	116	2,005	16	0.964	0.945	0.788	0.992
**G-CSF**	319	612	1,509	128	0.714	0.711	0.343	0.922
**Nivolumab**	17	157	1,964	430	0.038	0.926	0.098	0.820
**Ipilumumab**	NR	NR	NR	NR	< 0.02	> 0.99	0.714	0.827
**Irinotecan**	NR	NR	NR	NR	< 0.04	> 0.99	0.778	0.830
**Pegfilgrastim**	297	471	1,650	150	0.664	0.778	0.387	0.917
**Topotecan**	NR	NR	NR	NR	< 0.03	> 0.99	0.846	0.829
**Platinum**	441	1,603	NR	NR	0.987	0.244	0.216	0.989
**Etoposide or Pegfilgrastim**	434	528	1,593	13	0.971	0.751	0.451	0.992
**Etoposide or G-CSF**	435	659	1,462	12	0.973	0.689	0.398	0.992
**Etoposide or Irinotecan or Topotecan**	438	NR	2,001	NR	0.980	0.943	0.785	0.996
**Etoposide and no reported EGFR testing**	428	NR	2,017	19	0.957	0.951	0.805	0.991

NR, not reportable due to NCI privacy rules that prevent reporting cell sizes < 11 or counts that can be used to calculate cell sizes < 11. G-CSF includes both filgrastim and pegfilgrastim.

Agents that were promising but did not perform sufficiently well to replace etoposide in our final algorithms included pegfilgrastim (sensitivity = 41%, specificity = 94%, PPV = 39% and NPV = 94%), G-CSF (sensitivity = 71%, specificity = 71%, PPV = 34% and NPV = 92%), and platinum (sensitivity = 61%, specificity = 78%, PPV = 22% and NPV = 95%).

As a result of these investigations, and because irinotecan and topotecan are typically used as second-line therapy in SCLC, we finalized our candidate algorithms to the following two: 1) etoposide within 180 days of diagnosis and 2) etoposide plus no reported EGFR testing within 180 days of diagnosis.

### 3.2 Validation results

Both tested algorithms performed similarly, in the full cohort and in the systemic therapy subset (**Table 3**). In the full cohort, the algorithms had sensitivities between 54.3% and 55.8%, specificities of between 98.6% and 98.9%, PPVs of between 81.1% and 83.9%, and NPVs of between 95.4% and 95.5%. In the systemic therapy subset, the algorithms had sensitivities between 94.0% and 96.5%, specificities of between 95.2% and 96.1%, PPVs of between 81.1% and 83.9%, and NPVs of between 98.7% and 99.0%. Note that, because all algorithms are based on systemic therapies, the true positive and false positive counts in the full cohort and systemic therapy subset are identical, leading to identical PPVs. See **Supplementary Materials** for specific details on the algorithm for implementation.

**Table 3:** Validation results in the full cohort (N=23,934) and the systemic therapy subset (N=7,438).

Algorithm	True Positive	False Positive	True Negative	False Negative	Sensitivity	Specificity	PPV	NPV
Full Cohort (N = 23,934)								
**Etoposide**	1,251	280	21,379	1,024	0.550	0.987	0.817	0.954
**Etoposide and no reported EGFR testing**	1,236	237	21,422	1,039	0.543	0.989	0.839	0.954
Systemic Therapy Subset (N = 7,438)								
**Etoposide**	1,251	280	5,843	64	0.951	0.954	0.817	0.989
**Etoposide and no reported EGFR testing**	1,236	237	5,886	79	0.940	0.961	0.839	0.987

We conducted sensitivity analyses of these two algorithms by including all information from the 30-day period prior to the first lung cancer diagnosis (**Table 4**). The results were virtually identical to the primary results suggesting that the additional 30-day period added little to the algorithm.

**Table 4:** Sensitivity analysis results for including 30 days prior to diagnosis.

Algorithm	Population	True Positive	False Positive	True Negative	False Negative	Sensitivity	Specificity	PPV	NPV
Full Cohort (N = 23,934)									
**Etoposide**	Diagnosed	1,251	282	21,377	1,024	0.550	0.987	0.816	0.954
**Etoposide and no reported EGFR testing**	Diagnosed	1,235	238	21,421	1,040	0.543	0.989	0.838	0.954
Systemic Therapy Subset (N = 7,438)									
**Etoposide**	Treated	1,251	280	5,843	64	0.951	0.954	0.817	0.989
**Etoposide and no reported EGFR testing**	Treated	1,235	236	5,887	80	0.939	0.961	0.840	0.987

We conducted another sensitivity analysis in the population < 65 years of age (**Table 5**). In the younger population, the results were consistent with, but slightly different from, the results in the older population. Sensitivities were slightly higher, specificities were slightly lower, PPVs were slightly lower, and NPVs were slightly lower.

**Table 5:** Sensitivity analysis results for patients aged 19-65 years.

Algorithm	True Positive	False Positive	True Negative	False Negative	Sensitivity	Specificity	PPV	NPV
Full Cohort (N = 3,481)								
**Etoposide**	349	88	2,804	240	0.593	0.970	0.799	0.921
**Etoposide and no reported EGFR testing**	348	81	2,811	241	0.591	0.972	0.811	0.921
Systemic Therapy Subset (N = 1,186)								
**Etoposide**	349	88	740	NR	0.975	0.894	0.799	0.988
**Etoposide and no reported EGFR testing**	348	81	747	NR	0.972	0.902	0.811	0.987

## 4 Discussion

This cross-sectional study describes factors that could be used to identify SCLC patients among a cohort of lung cancer patients using claims datasets in the US. The factor most likely to be useful in making this distinction was the use of etoposide. Given the utility of using a systemic therapy to identify SCLC, it is important to note that more than 69% of all diagnosed lung cancer patients did not receive outpatient systemic therapy. This makes an etoposide-based algorithm useful for identifying SCLC patients treated with systemic therapy, but not for identifying SCLC patients not treated with systemic therapy.

The use of outpatient systemic therapy may be related to age, stage, frailty, and comorbidity burden. As has been shown in other studies, patients with a poor prognosis are less likely to receive outpatient systemic therapy (16–18). However, we should also note that our study included patients of all stages; therefore, some patients with early stage, with readily resectable cancers may also be less likely to receive systemic therapy.

The use of a single code for etoposide as part of first-line therapy shows promise for identifying a cohort of SCLC patients among all treated patients in this time period, based on its PPV of 82% and NPV of 99%. Translated into actual numbers, the algorithm was able to identify 1,251 SCLC patients (true positives) and missed only 64 SCLC patients (false negatives). The cohort also included 280 NSCLC patients (false positives) resulting in approximately 4.5 SCLC patients for each NSCLC patient. Adding the EGFR code improved accuracy by reducing the false positive count to 237, and increased the ratio to 5.25 SCLC patients for each NSCLC patient.

We designed this study to emulate a typical administrative claims data study design intended to study treated patients with SCLC by using a 12-month look-back period to capture comorbid conditions and to rule out prior lung cancer. In such a study, the cohort of treated patients would likely be identified using ICD-10-CM codes for lung cancer along with the use of medications typically used in SCLC within a period of time after diagnosis. Once treatment initiation is identified, patients might be followed for outcomes including hospitalization use, hospice use, emergency department use, and the cost of care. Using the etoposide algorithm would identify most SCLC patients and facilitate such an interrogation of routinely collected data.

Claims related to EGFR testing were somewhat useful as well. In theory few, if any, SCLC patients would receive testing for EGFR and other NSCLC-specific genetic variants. This would seem to be a promising way to rule out false positives (i.e., NSCLC patients who receive etoposide). However, HCPCS/CPT codes for this kind of testing are typically related to the testing method, and are not specific to the variant. Hence, they have limited utility in identifying EGFR-specific testing that patients underwent (19). However, to the extent that researchers have access to relevant coding, our analyses suggest that EGFR testing would be somewhat useful in reducing false positives (20).

Reliable subtyping of lung cancer is critical for optimal clinical decision making (21). However, our objective was not to predict lung cancer subtypes in routine clinical practice; rather, it was to enable researchers using claims databases, which lack other data elements to identify the various subtypes of lung cancer, to classify patients as SCLC versus NSCLC by using commonly available data elements. In addition, we elected to create a deterministic definition (i.e., each person’s SCLC status is either true or false) and not a probabilistic one because it makes it easier for researchers to determine whether our definition is appropriate for their research question.

As with any study using observational data, several limitations should be considered when interpreting these findings. Our focus was on patients who primarily received “first-line” outpatient systemic therapy (i.e., therapy within 180 days of diagnosis). Specific systemic therapy agents provided in the hospital setting were not identifiable; hence these patients were not included in the systemic therapy subset unless they also received outpatient therapy. Our US-based study population was limited to Medicare enrollees diagnosed during 2016 and 2017. These analyses may not reflect the most recent treatment trends due to the time lag, and they may vary outside of the US to the extent that other systemic therapies are used in first-line SCLC. However, the performance of the algorithm would not be anticipated to change as long as etoposide remains part of first-line SCLC treatment. Therefore, its utility is likely to be reasonably well-understood based on the relevant treatment guidelines for the years of data used in a claims-based study. We also did not evaluate the algorithm in subsets of patients according to the extent of their lung cancer (e.g., metastatic versus localized). Finally, these results do not suggest a possible way to identify untreated SCLC patients.

In conclusion, we developed and validated an algorithm to identify SCLC patients treated with outpatient systemic therapy with high accuracy within administrative claims datasets. Such an algorithm would be useful for evaluating treatment patterns and clinical outcomes of SCLC patients treated with associated outpatient systemic therapies.

## Data Availability

The datasets presented in this article are not readily available because this data contains potentially identifiable information. Researchers may request SEER-Medicare data from the National Cancer Institute. Requests to access the datasets should be directed to https://healthcaredelivery.cancer.gov/seermedicare.
