# Supplementary material for: Development of an algorithm to identify small cell lung cancer patients in claims databases
Source: Front Oncol. 2024 Aug 15;14:1358562. doi: 10.3389/fonc.2024.1358562 (PMC11357974; doi:10.3389/fonc.2024.1358562)
Supplement: Supplementary file 1 [file DataSheet1.pdf]

1. Supplementary tables

Supplementary Table 1: Baseline Demographic and Clinical Information for the Exploration and Validation Samples

Variable	Diagnosed Exploration	Treated Exploration	Diagnosed Validation	Treated Validation
	N = 7,978	N = 2,568	N = 23,934	N = 7,438
	% (N) or Mean (SD)	% (N) or Mean (SD)	% (N) or Mean (SD)	% (N) or Mean (SD)
**Age at Diagnosis**				
Age (years)	76.2 (7.0)	74.4 (5.9)	76.3 (7.0)	74.5 (6.0)
Age 66-69	18.9% (1,510)	24.1% (620)	18.9% (4,513)	24.0% (1,787)
Age 70-74	27.3% (2,176)	31.6% (811)	26.8% (6,418)	31.4% (2,339)
Age 75-79	24.1% (1,922)	25.2% (648)	23.8% (5,704)	24.4% (1,816)
Age 80-84	15.7% (1,252)	12.7% (325)	16.1% (3,843)	13.0% (966)
Age 85+	14.0% (1,118)	6.4% (164)	14.4% (3,456)	7.1% (530)
**Sex and Race Categories**				
Male	45.9% (3,663)	50.2% (1,289)	44.2% (10,585)	47.4% (3,525)
White	89.7% (7,153)	88.3% (2,267)	89.8% (21,493)	89.3% (6,643)
Black	5.6% (449)	5.8% (148)	5.8% (1,390)	5.8% (434)
Asian	4.0% (323)	5.2% (133)	3.6% (862)	4.1% (305)
Other	0.7% (53)	0.8% (20)	0.8% (189)	0.8% (56)
**Marital Status**				
Married	33.9% (2,707)	40.4% (1,038)	33.6% (8,040)	39.1% (2,905)
Widowed	15.4% (1,226)	13.2% (338)	15.0% (3,593)	12.4% (924)
Single / Divorced	14.9% (1,189)	14.2% (364)	14.7% (3,515)	15.0% (1,113)
Unknown	3.3% (263)	3.0% (77)	3.1% (741)	3.0% (222)
Missing	32.5% (2,593)	29.2% (751)	33.6% (8,045)	30.6% (2,274)
**Histology**				
Small Cell	9.1% (725)	17.4% (447)	9.5% (2,275)	17.7% (1,315)
Non-Small-Cell	90.9% (7,253)	82.6% (2,121)	90.5% (21,659)	82.3% (6,123)
**SEER Stage**				

Variable	Diagnosed Exploration	Treated Exploration	Diagnosed Validation	Treated Validation
	N = 7,978	N = 2,568	N = 23,934	N = 7,438
	% (N) or Mean (SD)	% (N) or Mean (SD)	% (N) or Mean (SD)	% (N) or Mean (SD)
In Situ	0.5% (39)	NR	0.5% (110)	NR
Local	34.3% (2,602)	9.8% (248)	34.0% (7,731)	9.5% (695)
Regional	23.5% (1,780)	32.3% (815)	23.8% (5,414)	32.3% (2,356)
Distant	41.7% (3,166)	57.8% (1,461)	41.6% (9,461)	58.1% (4,238)
Missing	4.9% (391)	NR	5.1% (1,218)	NR
**AJCC Stage**				
In Situ	0.5% (28)	NR	0.5% (83)	NR
Stage 1	32.7% (1,736)	7.4% (137)	33.1% (5,219)	7.5% (396)
Stage 2	8.2% (435)	10.3% (191)	7.6% (1,193)	8.9% (471)
Stage 3	18.0% (955)	29.2% (539)	18.2% (2,869)	29.7% (1,568)
Stage 4	40.6% (2,156)	53.0% (979)	40.7% (6,426)	53.9% (2,850)
Missing	33.4% (2,668)	NR	34.0% (8,144)	NR
**Year of Diagnosis**				
2016	57.4% (4,582)	51.8% (1,329)	57.2% (13,694)	51.0% (3,795)
2017	42.6% (3,396)	48.2% (1,239)	42.8% (10,240)	49.0% (3,643)
**Percent Poverty in Census Tract**				
Poverty 0-4%	20.5% (1,639)	20.8% (533)	21.1% (5,052)	21.0% (1,559)
Poverty 5-9%	25.3% (2,017)	26.1% (669)	24.5% (5,865)	24.4% (1,816)
Poverty 10-19%	25.7% (2,047)	25.1% (645)	25.5% (6,096)	26.7% (1,984)
Poverty 20%+	17.6% (1,402)	17.7% (455)	17.4% (4,157)	16.7% (1,241)
Missing	10.9% (873)	10.4% (266)	11.5% (2,764)	11.3% (838)
**Metropolitan Status of County**				
Metro Area ≥ 1 Million Pop.	52.9% (4,221)	53.0% (1,361)	52.7% (12,622)	52.0% (3,865)
Metro Area < 1 Million Pop.	25.6% (2,042)	26.6% (682)	25.2% (6,040)	27.0% (2,006)
Adjacent to Metro Area	9.3% (739)	9.7% (248)	9.0% (2,159)	9.4% (699)

Variable	Diagnosed Exploration	Treated Exploration	Diagnosed Validation	Treated Validation
	N = 7,978	N = 2,568	N = 23,934	N = 7,438
	% (N) or Mean (SD)	% (N) or Mean (SD)	% (N) or Mean (SD)	% (N) or Mean (SD)
Not Adjacent to Metro Area	6.0% (481)	6.2% (159)	6.2% (1,487)	6.4% (475)
Missing	6.2% (495)	4.6% (118)	6.8% (1,626)	5.3% (393)

2. Codes and algorithm implementation details

Etoposide algorithm:

For each patient, identify the first date of diagnosis of lung cancer. This is the diagnosis date (day 0). Identify the presence of any codes for etoposide on days 0 through 179. If any etoposide records are identified, classify the person as having small cell lung cancer. Otherwise, classify the person as having non-small cell lung cancer.

Etoposide plus no reported EGFR testing algorithm:

For each patient, identify the first date of diagnosis of lung cancer. This is the diagnosis date (day 0). Identify the presence of any codes for etoposide and for EGFR testing on days 0 through 179. If any etoposide records are identified and if no EGFR testing records are identified, classify the person as having small cell lung cancer. Otherwise, classify the person as having non-small cell lung cancer.

ICD-10-CM codes for lung cancer:
C34.00, C34.01, C34.02, C34.10, C34.11, C34.12, C34.2, C34.30, C34.31, C34.32, C34.80, C34.81, C34.82, C34.90, C34.91, C34.92

HCPCS code for EGFR testing:
81235

HCPCS codes for etoposide:
C9414, C9425, J9182, J8560, J9181

NDC codes for etoposide
00013733691
00013734694
00013735688
00013736673
00015306120
00015306124
00015306220

00015306224
00015308420
00015309145
00015309520
00015309530
00015309595
00015340420
00074148501
00074148502
00074148503
00143937601
00143951001
00143951101
00143951201
00186157131
00364302853
00378326632
00378326694
00703564301
00703564303
00703564601
00703564603
00703565301
00703565601
00703565691
00703565701
00703565791
00703565801
00703566701
00703566801
10019093001
10019093002
10019093035

10019093054
10130029101
10130029201
10130029301
16729011408
16729011411
16729011431
16729011432
16729026231
16729026232
42605003101
42605003201
42605003301
47351003725
47351003805
47351008050
50053071102
50053071408
51079096501
51079096505
51305006120
51305006124
51305006220
51305006224
51305008420
51305009520
51305009530
51305040420
51927277200
53905029101
54868535500
54868535501
54868535502

55361163904
55390029101
55390029201
55390029301
55390049101
55390049201
55390049301
55567005002
57423000104
57527010905
57884000101
57884000102
58406071112
58406071418
61126009520
61269041020
62180565301
62180565601
62180565701
63323010401
63323010405
63323010406
63323010425
63323010450
67108005002
67108340402
68001026522
68001026523
68001026524
68001026525
68001026526
68001026527
